# Derivation of Luminescent Mesoporous Silicon Nanocrystals from Biomass Rice Husks by Facile Magnesiothermic Reduction

**DOI:** 10.3390/nano11030613

**Published:** 2021-03-01

**Authors:** Sankar Sekar, Sejoon Lee

**Affiliations:** 1 Division of Physics & Semiconductor Science, Dongguk University-Seoul, Seoul 04620, Korea; sanssekar@dongguk.edu; 2 Quantum-Functional Semiconductor Research Center, Dongguk University-Seoul, Seoul 04620, Korea

**Keywords:** biomass rice husk, silicon, nanocrystals, luminescence, high porosity

## Abstract

High-quality silicon (Si) nanocrystals that simultaneously had superior mesoporous and luminescent characteristics were derived from sticky, red, and brown rice husks via the facile and cost-effective magnesiothermic reduction method. The Si nanocrystals were confirmed to comprise an aggregated morphology with spherical nanocrystals (e.g., average sizes of 15–50 nm). Due to the surface functional groups formed at the nanocrystalline Si surfaces, the Si nanocrystals clearly exhibited multiple luminescence peaks in visible-wavelength regions (i.e., blue, green, and yellow light). Among the synthesized Si nanocrystals, additionally, the brown rice husk (BRH)-derived Si nanocrystals showed to have a strong UV absorption and a high porosity (i.e., large specific surface area: 265.6 m^2^/g, small average pore diameter: 1.91 nm, and large total pore volume: 0.5389 cm^3^/g). These are indicative of the excellent optical and textural characteristics of the BRH-derived Si nanocrystals, compared to previously reported biomass-derived Si nanocrystals. The results suggest that the biomass BRH-derived Si nanocrystals hold great potential as an active source material for optoelectronic devices as well as a highly efficient catalyst or photocatalyst for energy conversion devices.

## 1. Introduction

Silicon (Si) is one of the most powerful semiconductors that have led to the strong advancement of modern electronics. However, bulk Si is inadequate as an active material (i.e., a core part for visible light emission or detection) in optoelectronic devices because of its indirect bandgap with an infrared energy gap of 1.12 eV at 300 K [1,2]. One effective way to overcome this issue is the nanocrystallization of Si, which can allow us to create visible light emission and detection characteristics, attributable to the quantum confinement effect and the size effect in Si nanocrystals [3,4,5,6,7]. Therefore, the fabrication of Si nanocrystals has attracted tremendous attention in wide scientific and technologic communities because of their vast application fields in optoelectronics as well as electronics. For instance, nanofloating gate flash memory devices [8,9,10,11], field-effect electroluminescence devices [12,13], tandem solar cells [14], and optical waveguides [15,16] are typical examples that can use the quantum-confined electronic energy system in Si nanocrystals. Owing to the high porosity of Si nanocrystals, furthermore, they are also very useful as an active source material in energy storage and conversion devices. For example, Si nanocrystals could be used as an effective catalyst for the hydrogen evolution reaction [17], and be utilized as an anodic source material for highly energy-efficient lithium-ion and sodium-ion batteries [18,19,20].

To obtain highly porous and/or highly luminescent Si nanocrystals, many researchers have contrived and designed various experimental methods, e.g., laser ablation [21], nonthermal plasma processes [22], pulsed laser deposition [23], chemical doping [24], electrochemical etching [25], chemical vapor deposition [26], annealing of borophosphosilicate glasses [27], and laser pyrolysis [28]. However, these methods require expensive equipment, complex procedures, and high thermal budgets (also see Table S1, Supplementary Materials). Therefore, a facile and cost-effective approach is necessary for the mass production of Si nanoparticles. Considering both the cost-effectiveness and the eco-friendliness, biomass wastes are truly fascinating resources that can provide us with natural siliceous constituents. Accordingly, various biomass resources (e.g., sugarcane bagasse [29], bamboo leaves [29,30], beach sand [31], corn leaves [32], and rice husks (RHs) [33,34,35,36,37]) were used in earlier studies for the derivation of high-quality Si nanocrystals (also see Table S2 for the comparison of Si production from various biomass resources by using several experimental techniques, Supplementary Materials). Among them, RHs are one of the most prominent siliceous precursors because of their huge availability and high silica contents [18,38,39]. These provide us with a good hint to produce a large amount of Si nanocrystals via the recycling of biomass RHs. Despite such benefits, to our best knowledge, the synthesis of RH-derived high-quality Si nanocrystals with both high porosity and high luminescence has not been reported to date. Furthermore, the coexistence of both mesoporous and luminescent characteristics in a single material system is truly helpful for future energy technology; for example, the photocatalytic hydrogen evolution reaction [40,41,42] and oxygen evolution reaction [43,44,45].

We, therefore, investigated the facile derivation of mesoporous-and-luminescent Si nanocrystals from various RHs (i.e., sticky RHs, red RHs, and brown RHs) through the magnesiothermic reduction process, which can be simply performed in an inert atmosphere without toxic gases and vacuum facilities. Herein, we report on a comprehensive study from the synthesis to the characterization of RH-derived mesoporous luminescent Si nanocrystals. The kinetics of magnesiothermic reduction for Si nanocrystal production is discussed, and the structural, morphological, optical, and textural properties of the synthesized Si nanocrystals are thoroughly examined in detail.

## 2. Experimental Section

### 2.1. Material Preparation

The biomass sticky rice husks (S-RHs) were collected from Gyeonggi, South Korea, and the red rice husks (R-RHs) and brown rice husks (B-RHs) were collected from Perambalur, Tamil Nadu, India. Hydrochloric acid (HCl, 37%), hydrofluoric acid (HF, 48%), and magnesium (Mg, 99% purity) powders were purchased from Sigma-Aldrich (St. Louis, MO, USA) and used with no additional purification.

### 2.2. Synthesis of Si Nanocrystals

The derivation of the Si nanocrystals via magnesiothermic reduction can be described by the following chemical reactions:

(1) $${\mathrm{SiO}}_{2}\left(\mathrm{Ashes}\right)+\mathrm{HCl}\underset{27\;{}^{\circ}C\;\left(2\;h\right)}{\to }{\mathrm{SiO}}_{2}\left(\mathrm{Colloidal}\right)+H_{2}O+\mathrm{MCl}$$

(2) $${\mathrm{SiO}}_{2}\left(\mathrm{Colloidal}\right)\underset{700\;{}^{\circ}C\;\left(2\;h\right)}{\to }{\mathrm{SiO}}_{2}\left(\mathrm{Nanoparticles}\right)$$

(3) $${\mathrm{SiO}}_{2}\left(\mathrm{Nanoparticles}\right)+2\mathrm{Mg}\underset{700\;{}^{\circ}C\;\left(2\;h\right)}{\to }\mathrm{Si}\left(\mathrm{Nanocrystals}\right)+2\mathrm{MgO}$$

(4) $$\mathrm{Si}\left(\mathrm{Nanocrystals}\right)+2\mathrm{MgO}+4\mathrm{HCl}\underset{27\;{}^{\circ}C\;\left(10\;h\right)}{\to }\;\mathrm{Si}\;\left(\mathrm{Nanocrystals}\right)+2{\mathrm{MgCl}}_{2}+2H_{2}O$$

**Figure 1:**
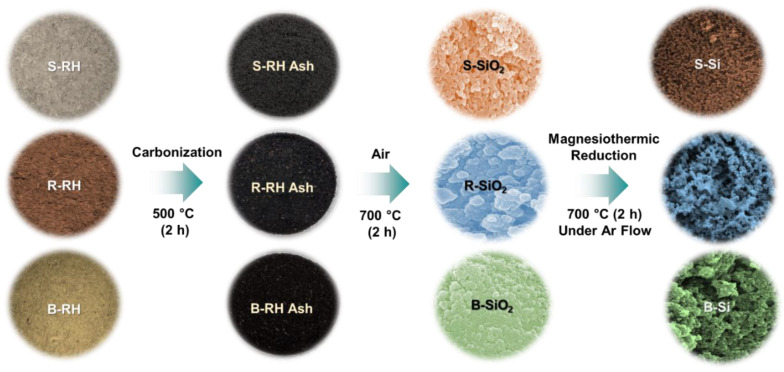
Schematic illustration of the magnesiothermic reduction process for synthesizing the Si nanocrystals by using biomass sticky rice husks (S-RHs), red rice husks (R-RHs), and brown rice husks (B-RHs).

M in Equation (1) is the possible precipitates from raw bio-silica in the biomass RHs (e.g., Na, K, Ca, Fe, and Mg), which are normally removed as MCl after the HCl treatment. To investigate the dependence of the biomass RH resources, we used three different types of RHs, i.e., S-RHs, R-RHs, and B-RHs. As schematically illustrated in Figure 1, initially, all three types of RHs were carbonized at 500 °C for 2 h under an air atmosphere to obtain their ashes. Then, 3 g of each RH ash was stirred in a 10% HCl solution for 2 h to eliminate metal ions and contamination (e.g., Equation (1)). After HCl leaching, the samples were rinsed with deionized water (DI), filtered, and dried at 150 °C for 15 h in an electric oven. Subsequently, the samples were transferred to an alumina crucible and were further calcinated at 700 °C for 2 h under an air atmosphere in a muffle furnace (e.g., Equation (2)). During this calcination step, the silica nanopowders were obtained from the HCl-leached RH ashes. Next, the SiO_2_ nanopowders were reduced into the Si nanocrystals through magnesiothermic reduction. To achieve the reduction reaction, as a primary task, each type of SiO_2_ nanopowder (2 g) was mixed with the Mg powders (0.5 g). Then, the mixture powders were annealed at 700 °C for 2 h under an Ar atmosphere in a tube furnace (e.g., Equation (3)). The obtained products were stimulated with 1 M HCl (HCl:H_2_O:EtOH = 0.66:4.72:8.88 molar ratio) for 10 h to remove MgO (e.g., Equation (4)). After the HCl treatment, the colloidal solutions were reacted with 5% HF for 1 h to eliminate the residual SiO_2_ inside the magnesiothermically reduced Si nanopowders. Finally, the obtained Si nanopowders were washed in DI water, filtered, and dried at 80 °C for 12 h under vacuum. Through these sequences, we were able to obtain the powder type of the Si nanocrystals. For convenience, we denote the three different types of the Si nanocrystals as S-Si, R-Si, and B-Si, which were derived from S-RHs, R-RHs, and B-RHs, respectively.

### 2.3. Characterization of Material Properties

The morphological and the compositional properties of the Si nanocrystals were monitored by field-emission scanning electron microscopy (FE-SEM) using an Inspect F50 system (FEI Co., Mahwah, NJ, USA) and its in situ energy dispersive X-ray (EDX) spectroscopy, respectively. The structural and the vibrational properties of the samples were characterized by Raman scattering spectroscopy using a LabRAM HR800 system (HORIBA Jobin Yvon Inc., Edison, NJ, USA) and X-ray diffractometry (XRD) using a D2 Phaser system (Bruker, Madison, WI, USA), respectively. The functional groups of the nanocrystals were examined by Fourier transform infrared (FTIR) spectroscopy using a Spectrum-100 system (Perkin Elmer, Shelton, CT, USA). The optical absorption and emission characteristics were evaluated by UV–VIS spectroscopy using an S-3100 system (Scinco, Seoul, Republic of Korea) and photoluminescence (PL) spectroscopy using a Cary Eclipse Fluorescence Spectrophotometer (Agilent Technologies, Santa Clara, CA, USA), respectively. The textural properties were analyzed by nitrogen absorption–desorption isotherms (N_2_-ADI) using a BELSORP-mini II system (MicrotracBEL, Osaka, Japan). 

## 3. Results and Discussion

### 3.1. Morphological and Compositional Properties

**Figure 2:**
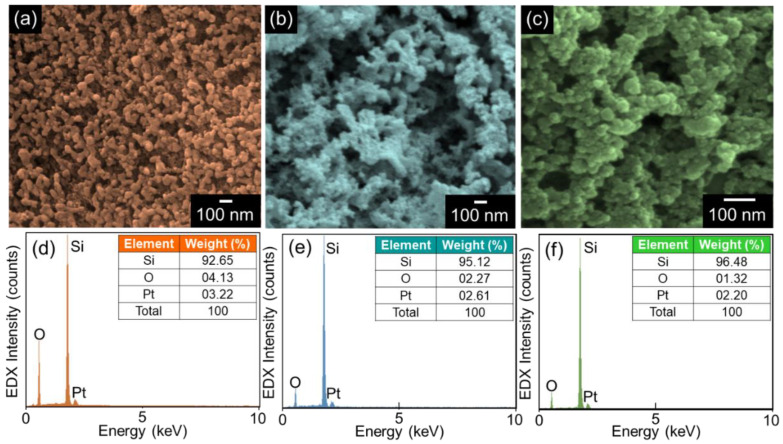
Field-emission scanning electron microscopy (FE-SEM) images of the (**a**) S-Si, (**b**) R-Si, and (**c**) B-Si nanocrystals and energy dispersive X-ray (EDX) spectra of the (**d**) S-Si, (**e**) R-Si, and (**f**) B-Si nanocrystals. The inset in each EDX graph summarizes the compositional properties of the Si nanoparticles.

Figure 2 shows the FE-SEM images of the S-Si, R-Si, and B-Si nanocrystals. The S-Si sample exhibited cylindrically interconnected spherical nanocrystals (Figure 2a). However, the R-Si and the B-Si samples displayed a nanosponge-like morphology, where a lot of small spherical nanocrystals were densely aggregated (Figure 2b,c).

Here, it should be noticed that the average crystal size of B-Si (~15 nm) is much smaller than those of R-Si (~35 nm) and S-Si (~50 nm). We believe such a discrepancy is attributable to the smaller contents of Si species in the raw sources of the B-RH ashes (Si~2.46%) than the R-RH (Si~5.95%) and S-RH ashes (Si~24.62%) (see Figure S1, Supplementary Materials). In other words, during the acid treatment and the calcination step (i.e., Equations (1) and (2)), the size of the colloidal SiO_2_ should be smaller for the B-RH case than the others because the lower quantity of Si species in B-RH (i.e., raw bio-silica in the biomass resource) may increase the segregation of the silica nanoparticles [46]. According to previous literature [47,48], using the Fokker–Planck equation [49,50,51], the size distribution of the Si nanoparticles (*C*(*i,t*)) can be described as follows:

(5) $$\frac{\partial C\left(i,t\right)}{\partial t}=-\left(N\left(t\right)-N_{e}\right)\frac{\partial \left(k\left(i\right)C\left(i,t\right)\right)}{\partial i}+\left(N\left(t\right)+N_{e}\right)\frac{\partial^{2}\left(k\left(i\right)C\left(i,t\right)\right)}{\partial^{2}i^{2}},$$

where *i* is the number of Si atoms, *N*_e_ is the equilibrium concentration of impurity atoms in the substance matrix, and *N*(*t*) is the number of Si atoms in the nanocrystal at each moment of time. The kinetic coefficient *k*(*i*) in Equation (5) is proportional to both the diffusion coefficient of the Si atoms in the matrix *D* and the Si nanocrystal radius (*R*(*i*)):

(6) k(i)=4πDR(i)

(7) $$R\left(i\right)=b{\left(i+m\right)}^{\alpha },$$

where *b* is the distance parameter of the nanocrystals, *m* is the size homogeneity factor, and *α* is the geometry factor (= 1/3 for spherical nanocrystals). Hence, the smaller size of B-Si can be interpreted as resulting from the lower concentration of Si species in B-RH. We therefore conjecture that the size of the Si nanocrystals could be automatically controlled by choosing the type of the biomass raw resources.

Next, the compositional properties of the samples were evaluated by EDX. As shown in Figure 2d–f, all the prepared samples were composed of the main species of Si and O, arising from the body and the surface of the nanocrystals, respectively. The additional component of Pt is thought of as sprouting from the conductive coating layer for the FE-SEM measurements.

### 3.2. Structural and Vibrational Properties

**Figure 3:**
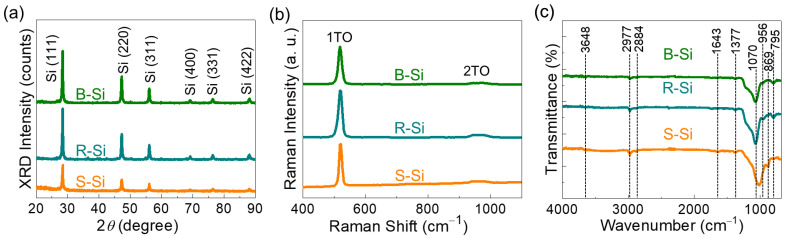
(**a**) X-ray diffractometry (XRD) patterns, (**b**) Raman spectra, and (**c**) Fourier transform infrared (FTIR) spectra of the S-Si, R-Si, and B-Si nanocrystals.

The crystallographic properties of the S-Si, R-Si, and B-Si samples were characterized by XRD. As shown in Figure 3a, all of the three samples exhibited the typical diffraction patterns of crystalline Si at 28.4°, 47.4°, 56.1°, 69.1°, 76.4°, and 88.2°, which correspond to the (111), (220), (311), (400), (331), and (422) Si planes (JCPDS No. 27-1402 [18,33,52,53]), respectively. This means that all three, S-Si, R-Si, and B-Si, were well crystallized via magnesiothermic reduction from the biomass resources of the S-RH, R-RH, and B-RH ashes, respectively. By using the Scherer formula [54,55,56], the average crystallite sizes of the S-Si, R-Si, and B-Si nanocrystals were determined to be 33, 28, and 22 nm, respectively. This corroborates the dependence of the Si nanocrystal size on the kind of biomass raw source; i.e., the Si nanocrystal size relies on the different Si contents in each RH, as confirmed in Figure 2. The nanocrystallization of the samples was further elucidated by Raman spectroscopy measurements. As shown in Figure 3b, the Raman spectra of all three samples revealed a similar feature of the typical Raman vibration modes from crystalline Si. Namely, the sharp peak at 519 cm^−1^ (i.e., the first-order transversal optical (TO) mode [18,57,58]) and the broad hump at 957 cm^−1^ (i.e., the second-order TO mode [18,57,58]) are clearly observable in all the samples, while no other Raman bands are visible. This demonstrates that the high-purity Si nanocrystals were effectively derived from the biomass RHs through the magnesiothermic reduction process. 

For the nanostructured materials, the functional groups of the elemental species and molecular states depend on the shape and the size of the nanomaterials because they rely on the bonding states at the surface terminals. To examine the functional groups of the samples, FTIR measurements were carried out. As shown in Figure 3c, the samples displayed several FTIR features at 795, 869, 956, 1070, 1377, 1643, 2884, 2977, and 3648 cm^−1^, all of which are closely relevant to the Si nanostructure. In other words, the transmission band at 795 cm^−1^ is ascribed to the Si–C stretching mode [36], and the vibrational band at 869 cm^−1^ is attributed to the Si–N stretching mode [59,60]. Similarly, the band at 956 cm^−1^ arose from the Si–H bending mode [61]. Additionally, the bands at 1070, 1377, 2884, and 2977 cm^−1^ correspond to the Si–O bending, CH_3_ bending, symmetric CH_2_ vibration, and CH_3_ stretching modes, respectively [59,62,63]. The bands at 3648 and 1643 cm^−1^ are responsible for the Si–OH vibrations [64]. 

### 3.3. Optical Properties

**Figure 4:**
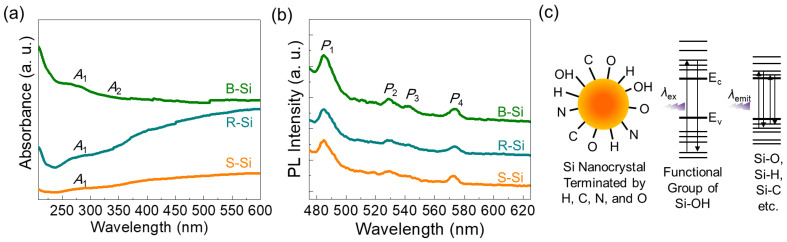
(**a**) UV-VIS absorption spectra, (**b**) photoluminescence (PL) spectra, and (**c**) light emission mechanism of the rice husk (RH)-derived biomass Si nanocrystals.

Figure 4a shows the UV−VIS absorption spectra of the S-Si, R-Si, and B-Si samples. The B-Si nanocrystals revealed strong UV adsorption, while the S-Si and the R-Si nanocrystals exhibited predominant visible-light adsorption characteristics. For B-Si, particularly, two distinct Si nanocrystal-related absorption bands are observable at *A*_1_~270 nm and *A*_2_~340 nm. Namely, the *A*_1_ peak and the *A*_2_ shoulder are associated with the *L*−*L* and the *Γ*−*Γ* transitions in nanocrystalline Si, respectively [6,36]. Since both the *A*_1_ and *A*_2_ adsorption intensities indicate the degree of nanocrystallization [65], one can observe that the B-Si sample was well crystallized with a smaller size than the others, as confirmed by FE-SEM and XRD.

**Figure 5:**
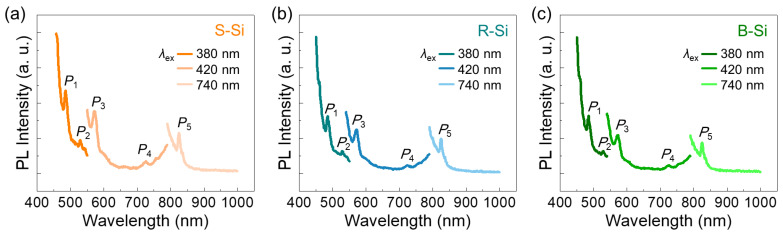
Excitation-dependent PL spectra of the (**a**) S-Si, (**b**) R-Si, and (**c**) B-Si nanocrystals.

According to earlier literature [3,4,5], those transitions originate from the discrete energy states within the modified electronic band structure of the Si nanocrystal. In other words, when the nanocrystal size becomes smaller than the exciton Bohr radius (~4 nm for Si), the subbands above the conduction band and below the valence band could be altered because of the quantum confinement effect. Then, discrete energy states would be created inside the modified electronic band structure. Furthermore, since the Si nanocrystal surface is typically terminated by H, C, and O (also see the FTIR for our samples in Figure 3c), the overlap of electron and hole wave-functions would become significant [65]. These subband modulation effects can be elucidated by PL. As shown in Figure 4b, the samples emitted visible light at *P*_1_~485 nm, *P*_2_~530 nm, *P*_3_~545 nm, and *P*_4_~573 nm. The strong *P*_1_ emission is reported to emerge from the radiative optical transitions between the discrete energy states that are created at the Si-OH surface functional groups [66] (also see Figure 4c). The other peaks at *P*_2_, *P*_3_, and *P*_4_ are also well known to arise from the radiative optical transitions between the energy states that are created by the surface functional groups of Si–O [67], Si–C [5], and Si–H [68,69], respectively. Figure 5 shows the excitation-dependent PL spectra of the S-Si, R-Si, and B-Si samples. As the excitation wavelength (*λ*_ex_) increased, the peak position of the light emission (*λ*_emit_) tended to shift to the longer wavelength region (i.e., red shift). Such a *λ*_emit_ dependence on *λ*_ex_ was present in all the samples, and the *λ*_emit_ positions were almost identical, regardless of the raw source of the RHs. These findings depict that the PL emission in all the samples originated from the surface functional group-related subband modulation rather than the quantum confinement effect.

### 3.4. Textural Properties

**Figure 6:**
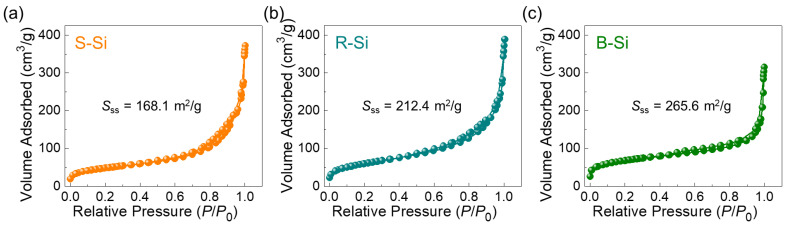
Nitrogen absorption–desorption isotherm (N_2_-ADI) characteristics of the (**a**) S-Si, (**b**) R-Si, and (**c**) B-Si nanocrystals.

The nanocrystallization of Si would have sturdily affected the porosity of the entire material system because the locally crystallized small nanocrystal has a high surface-to-volume ratio. In short, the nanocrystals must form structural voids at the surface area, giving rise to an increase in the porosity of the material. To assess the porosity of the S-Si, R-Si, and B-Si samples, thus, the textural characteristics were evaluated by the Brunauer–Emmett–Teller (BET) and the Barrett–Joyner–Halenda (BJH) analysis methods. Firstly, the specific surface area (*S*_ss_) was determined by N_2_-ADI measurements. As shown in Figure 6, all the samples exhibited Type-IV isotherm curves (classified according to IUPAC), representing the distinctive mesoporous characteristics of the materials [33,70]. Through the BET analysis, the *S*_ss_ values of the S-Si, R-Si, and B-Si nanocrystals were calculated to be 168.1, 212.4, and 265.6 m^2^/g, respectively. 

**Figure 7:**
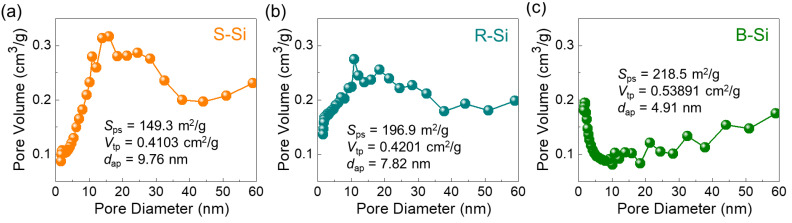
Pore size characteristics of the (**a**) S-Si, (**b**) R-Si, and (**c**) B-Si nanocrystals.

Next, the pore distribution characteristics were examined by BJH measurements (Figure 7). The pore surface areas (*S*_ps_) were determined to be 149.3, 196.9, and 218.5 m^2^/g for the S-Si, R-Si, and B-Si nanocrystals, respectively, and the total pore volumes (*V*_tp_) were calculated to be 0.4103, 0.4201, and 0.5389 cm^3^/g for S-Si, R-Si, and B-Si, respectively. Compared to S-Si and R-Si, the B-Si sample had larger magnitudes of *S*_ps_ and *V*_tp_ because of both the smaller nanocrystal size and the uniform distribution. Accordingly, the average pore diameter of B-Si (*d*_ap_~4.91 nm) was also smaller than those of S-Si (*d*_ap_~9.76 nm) and R-Si (*d*_ap_~7.82 nm). 

Finally, we compared the textural and the optical characteristics of our samples with various biomass-derived Si nanocrystals previously reported. As can be confirmed from Table 1 and Table 2, the B-Si nanocrystals possessed a superior porosity compared to the others, and had a great potential for multiple light emissions. Based on all the above results, therefore, one can surmise that the magnesiothermically reduced biomass B-Si nanocrystals hold great promise in various applications such as energy storage/conversion devices and optoelectronic devices.

## 4. Conclusions

High-quality Si nanocrystals that simultaneously showed both strong luminescence and high porosity were successfully derived from various RHs through the facile magnesiothermic reduction method. Owing to the different quantities of raw bio-silica in each RH, the size of the Si nanoparticles could be automatically varied from 15 to 50 nm. Due to the existence of the surface functional groups at the nanocrystals, the samples showed multiple light emissions in the visible-wavelength regions (i.e., blue, green, and yellow). Among the prepared samples, the B-Si nanocrystals exhibited a higher UV absorption and a superior porosity. The results depict that the B-RH-derived Si nanocrystals can play a crucial role as high-performance electrocatalysts, photocatalysts, and light emitters.

## Figures and Tables

**Table 1:** Comparison of the luminescence characteristics for various biomass-derived Si nanostructures.

Biomass	Nanostructures	Light Emission Color	References
Brown Rice Husks	Spherical Si Nanocrystals	Blue, Green, Yellow	This Work
Red Rice Husks	Spherical Si Nanocrystals	Blue, Green, Yellow	This Work
Sticky Rice Husks	Spherical Si Nanocrystals	Blue, Green, Yellow	This Work
Rice Husks	Si Nanoparticles	Green	[36]
Wheat Straws	Si Nanoparticles	Blue	[37]
Sugarcane Bagasse	Si Nanoparticles	Blue	[37]
Rice Husks	Si Nanoparticles	Blue	[37]

**Table 2:** Comparison of the pore characteristics for various biomass-derived Si nanostructures.

Biomass	*S*_ss_ (m^2^/g)	*V*_tp_ (cm^3^/g)	References
Brown Rice Husks	265.6	0.5389	This Work
Red Rice Husks	212.4	0.4201	This Work
Sticky Rice Husks	168.1	0.4103	This Work
Bamboo Leaves	302.13	0.526	[30]
Beach Sand	323	-	[31]
Corn Leaves	64	-	[32]
Rice Husks	288.4	0.35	[53]
Rice Husks	47.3	0.18	[34]
Rice Husks	127.05	0.2306	[71]
Waste Glass Microfiber Filter	66.12	0.64	[72]
Rice Husks	57.9	-	[73]

## Supplementary Materials

The Supplementary Materials are available online at https://www.mdpi.com/2079-4991/11/3/613/s1: Chemical Composition of Rice Husk Ashes; Comparison of Various Methods for Silicon Production; Figure S1. EDX spectra of (a) S-RH, (b) R-RH, and (c) B-RH ashes. Note that Pt in each raw source material arose from the conductive coating of Pt for better focusing and imaging during SEM and EDX measurements, Table S1. Summary of silicon synthesized from various resources through several experimental methods, Table S2. Summary of silicon synthesized from various biomass resources through several experimental methods.
