# Supplementary material for: Derivation of Luminescent Mesoporous Silicon Nanocrystals from Biomass Rice Husks by Facile Magnesiothermic Reduction
Source: Nanomaterials (Basel). 2021 Mar 1;11(3):613. doi: 10.3390/nano11030613 (PMC7999164; doi:10.3390/nano11030613)
Supplement: Supplementary file 1 [file nanomaterials-11-00613-s001.pdf]

nanomaterials

Supplementary Materials

Derivation of Luminescent Mesoporous Silicon Nanocrystals from Biomass Rice Husks by Facile Magnesiothermic Reduction

Sankar Sekar [1,2] and Sejoon Lee [1,2,*]

[1] Division of Physics & Semiconductor Science, Dongguk University-Seoul, Seoul 04620, Korea; sanssekar@dongguk.edu
[2] Quantum-functional Semiconductor Research Center, Dongguk University-Seoul, Seoul 04620, Korea
* Correspondence: sejoon@dongguk.edu

- **Chemical Composition of Rice Husk Ashes**

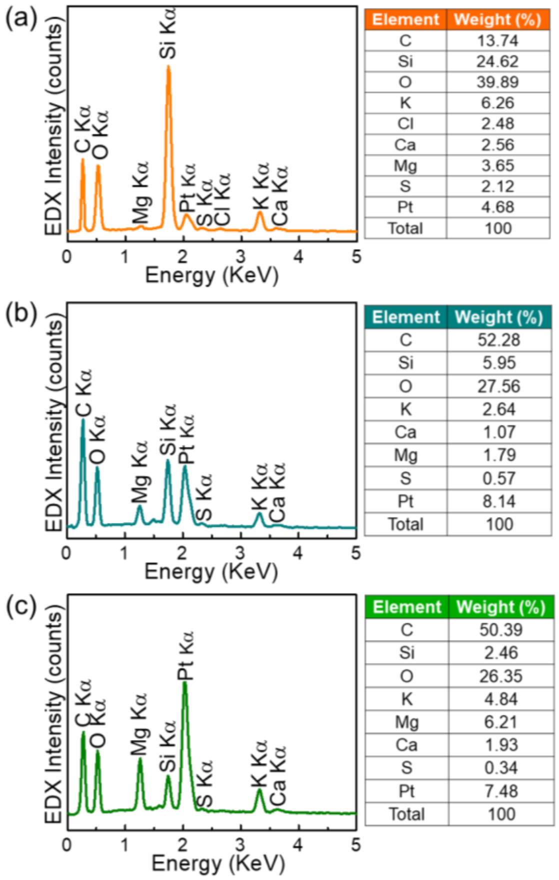

(a)

Element	Weight (%)
C	13.74
Si	24.62
O	39.89
K	6.26
Cl	2.48
Ca	2.56
Mg	3.65
S	2.12
Pt	4.68
Total	100

(b)

Element	Weight (%)
C	52.28
Si	5.95
O	27.56
K	2.64
Ca	1.07
Mg	1.79
S	0.57
Pt	8.14
Total	100

(c)

Element	Weight (%)
C	50.39
Si	2.46
O	26.35
K	4.84
Mg	6.21
Ca	1.93
S	0.34
Pt	7.48
Total	100

Figure S1. EDX spectra of (**a**) S-RH, (**b**) R-RH, and (**c**) B-RH ashes. Note that Pt in each raw source material arose from the conductive coating of Pt for better focusing and imaging during SEM and EDX measurements.

• Comparison of Various Methods for Silicon Production

Table S1. Summary of silicon synthesized from various resources through several experimental methods.

Method	Need for High-Vacuum Facility?	Precursor Materials	Hazardousness	Thermal Budget (Temperature, Power, etc.)
Laser Ablation	Yes	Silicon Bulks or Powders (Solid Phase)	No	High Power Excimer Laser (>a few J/cm^2)
Plasma Process	Yes	SiH_4, $SiCl_4$, etc. (Gas Phase)	Yes	RF Power (a few tens to hundreds W)
Pulsed Laser Deposition	Yes	Si or SiO_2 Ceramic Targets (Solid Phase)	No	High Power Excimer Laser (>a few J/cm^2)
Chemical Vapor Deposition	Yes	SiH_4, $SiCl_4$, etc. (Gas Phase)	Yes	High Temperature (~1000 °C)
Thermal Annealing	Yes	Siliceous Sources (Solid or Gas Phases)	Dependent on Precursors	Moderate Temperature (~500 °C)
Chemical Doping	Yes	Siliceous Sources (Solid and Liquid Phases)	Dependent on Precursors	Medium Temperature (700–800 °C)
Electrochemical Etching	No	Silicon Bulks or Powders (Solid Phase)	No	High Current Density (a few A/cm^2 for 1 min)
Molten-Salt Process	No	Every Siliceous Source and $CaCl_2$ (Solid Phase)	No	Medium–High Temperature (900–1000 °C)
Magnesiothermic Reduction	No	Every Siliceous Source and Mg (Solid Phase)	No	Medium Temperature (700–800 °C)

Table S2. Summary of silicon synthesized from various biomass resources through several experimental methods.

Study	Resources	Synthesis Method	Summary of Process Steps	Results
Z. Favors et al. [1]	Beach Sand	Magnesiothermic Reduction	· Calcination of sand at 900 °C in air<br>· HCl, HF, and NaOH leaching<br>· Ultrasonication for 1 h<br>· NaCl was mixed with SiO_2 (10:1 wt.%); then, the mixture was ultrasonicated for 4 h<br>· SiO_2:NaCl (1:0.9 wt.%) with Mg powder<br>· Transferred to swagelok-type reactors<br>· Annealed at 700 °C for 6 h in Ar-filled glovebox<br>· HCl (5 M) and HF (10%) acid etching	· Porous network of interconnected crystalline silicon nanoparticles with high specific surface area of 323 $m^2 g^{-1}$
M. Sakamoto et al. [2]	Rice Husks	Pulsed Laser Melting	· HCl acid leaching<br>· RH annealed at 700 °C for 4 h by flowing O_2 in furnace<br>· SiO_2 mixed with Mg powder<br>· Annealed at 650 °C for 2 h under H_2/Ar gas<br>· HCl:Ethanol:H_2O (1.5:10:5) etching<br>· Nd:YAG laser (λ = 532 nm) was used as an energy source for melting processes<br>· Repetition rate: 10 Hz<br>· Irradiation time: 20 min<br>· Laser fluence: 50, 150, and 250 mJ/cm^2	· Nanocoral Si spheroidal structure with a particle size of ~200 nm<br>· When increasing the laser fluence (50 to 250 mJ/cm^2 pulse), the specific surface area of the Si nanoparticle was decreased (57.9 to 20.7 $m^2 g^{-1}$).
J.-H. Choi et al. [3]	Rice Husks	Molten-Salt Process	· Acid leaching and thermal process of RH<br>· RH-SiO_2 was mixed with NiO (20:1 at.%) in ethanol<br>· Polyvinyl alcohol and zinc stearate were added as binders<br>· Powders pressed at 100 bar in cylindrical mold<br>· Sintering at 1200 °C in air for 5 h<br>· Annealing of $CaCl_2$ at 850 °C in Ar atmosphere<br>· RH-SiO_2 + NiO pellet was wrapped in nickel mesh<br>· Electrodeoxidation was performed at 2.7–2.9 V for 0–10 h<br>· HCl (0.1 M) and HF (2%) etching	· Crystalline Si nanowires with diameter of ~300 nm and length of ~1 μm<br>· Si nanowires had excellent cycling and power performance in LIB anodes

A. Su et al. [4]	Corn Leaves	Aluminothermic Reduction	· Annealed at 650 °C in air for 3 h<br>· HCl (1 M) leaching for 12 h<br>· SiO_2 was mixed with Al powder and $AlCl_3$ powder<br>· Annealed at 250 °C for 12 h under Ar atmosphere	· Crystalline silicon nanoparticles with specific surface area of 64 m^2g^{-1}<br>· Si nanoparticles exhibited excellent long-term cycling and high rate capability in LIB anodes
S. Praneetha et al. [5]	Rice Husks	Microwave-Assisted Metallothermic Reduction	· HCl acid leaching<br>· Powder transferred to swagelok-type reactors<br>· Microwave solid-state process at 650 °C for 30 min<br>· Operated frequency at 2.45 GHz<br>· HCl:Ethanol:H_2O (19.3:172.6:28.3 mL) etching<br>· Stirring for 6 h<br>· Centrifuged and washed with water and ethanol, and dried in vacuum oven	· Interconnected nanoporous wall structure of Si with a wall thickness of ~23 nm and a pore diameter of 50–80 nm<br>· It was used as a suitable material for LIB anodes.
Present Work	Rice Husks (3 Types of RHs)	Magnesiothermic Reduction	· Carbonized at 500 °C for 2 h in air<br>· HCl leaching<br>· Incineration at 700 °C for 2 h in air<br>· SiO_2 mixed with Mg powder<br>· Incineration at 700 °C for 2 h in Ar atmosphere<br>· HCl (HCl:H_2O:EtOH = 0.66:4.72:8.88 molar ratio) etching for 10 h<br>· HF acid etching<br>· DI washing and drying	· Crystalline nature of spherical Si nanoparticles with average particle sizes of 15–50 nm<br>· High surface area of 265.6 m^2 g^{-1} and high porosity<br>· Light absorption near the UV region<br>· Blue, green, and yellow emissions<br>· The Si nanocrystals possess both high porosity and high luminescence/absorbance, which is indicative of great potential for highly efficient photocatalytic applications

References

1. Favors, Z.; Wang, W.; Bay, H.H.; Mutlu, Z.; Ahmed, K.; Liu, C.; Ozkan, M.; Ozkan, C.S. Scalable Synthesis of Nano-Silicon from Beach Sand for Long Cycle Life Li-ion Batteries. *Sci. Rep.* **2014**, *4*, 5623.
2. Sakamoto, M.; Terada, S.; Mizutani, T.; Saitow, K.-I. Large Field Enhancement of Nanocoral Structures on Porous Si Synthesized from Rice Husks. *ACS Appl. Mater. Interfaces* **2021**, *13*, 1105–1113.
3. Choi, J.-H.; Kim, H.-K.; Jin, E.-M.; Seo, M.W.; Cho, J.S.; Kumar, R.V.; Jeong, S.M. Facile and scalable synthesis of silicon nanowires from waste rice husk silica by the molten salt process. *J. Hazard. Mater.* **2020**, *399*, 122949.
4. Su, A.; Li, J.; Dong, J.; Yang, D.; Chen, G.; Wei, Y. An Amorphous/Crystalline Incorporated Si/SiO_x Anode Material Derived from Biomass Corn Leaves for Lithium-Ion Batteries. *Small* **2020**, *16*, 2001714.
5. Praneetha, S.; Murugan, A.V. Development of Sustainable Rapid Microwave Assisted Process for Extracting Nanoporous Si from Earth Abundant Agricultural Residues and Their Carbon-based Nanohybrids for Lithium Energy Storage. *ACS Sustain. Chem. Eng.* **2015**, *3*, 224–236.
